# Correlation Between the Gut Microbiome and Immunotherapy Response in Inflammatory Bowel Disease: A Systematic Review of the Literature

**DOI:** 10.7759/cureus.16808

**Published:** 2021-08-01

**Authors:** Katarzyna Karpinska-Leydier, Jashvini Amirthalingam, Khadija Alshowaikh, Anuruddhika Iroshani Jayarathna, Divya Bala Anthony Manisha R Salibindla, Gokul Paidi, Huseyin Ekin Ergin

**Affiliations:** 1 Internal Medicine, California Institute of Behavioral Neurosciences & Psychology, Fairfield, USA; 2 Neurology, California Institute of Behavioral Neurosciences & Psychology, Fairfield, USA; 3 Obstetrics and Gynecology, California Institute of Behavioral Neurosciences & Psychology, Fairfield , USA; 4 Pathology, California Institute of Behavioral Neurosciences & Psychology, Fairfield, USA; 5 General Practice, California Institute of Behavioral Neurosciences & Psychology, Fairfield, USA

**Keywords:** inflammatory bowel disease, crohn’s disease (cd), gastrointestinal microbiome, intestinal microbiota, gut flora, immune modulation therapy, ulcerative colitis (uc)

## Abstract

Inflammatory bowel disease (IBD) is an autoimmune disease associated with dysbiosis within the gastrointestinal tract. Characteristic taxonomic shifts of microbial populations are observed in disease progression and remission; however, despite similarities, there are many differences among individuals presenting with IBD including IBD subset, clinical course, and response to therapy. Much is still unknown about how these taxonomic shifts interact with immunotherapy and how genetic variants contribute. In this systematic review, we aimed to compile information on the interactions of the gut microbiome with immunotherapy in the course of disease and treatment of IBD patients. This systematic review was conducted as per the Preferred Reporting Items for Systematic Reviews and Meta-Analyses (PRISMA) guidelines and the PubMed database was methodically screened for literature search including keywords and Medical Subject Headings (MeSH) terms for relevant articles.

The quality appraisal was completed using the Cochrane Tool, Newcastle-Ottawa checklist, and the Scale for the Assessment of Narrative Review Articles (SANRA) checklist, as appropriate, and 11 relevant articles were included in this systematic review. Our review concludes that although there are characteristic taxonomic shifts between diseased and healthy patients, genetic variants are an important consideration in the predictive quality of disease and treatment decisions. The comparison between interactions of microbial populations and treatment in addition to the role of genetic variants may provide insight into treatment non-responders. Due to our limitations in current knowledge including the complexity of the microcosm, ethnic genetic variations among human populations, and our focus on relevant articles published in English over the past six years, we may have missed relevant studies. Future studies should focus on the comparison between Western and other cultural populations as well as further implementation of Genome-Wide Association Studies (GWAS) in clinical predictability.

## Introduction and background

One of the major functional components of the gastrointestinal system is the delicately balanced microbiota within it. Dysbiosis, the unfavorable alteration of this commensal, is linked to several human pathologies [1,2]. This imbalance has been implicated in chronic inflammation, including autoimmune disease in the form of inflammatory bowel disease (IBD) [3]. The two subsets of IBD are different in that ulcerative colitis (UC) continuously affects the large intestine and rectum with epithelial inflammation and superficial ulcers, while Crohn’s disease (CD) unevenly affects both the large and small intestines with full-thickness ulceration [3,4]. The understanding of the microbiome and its role in human health largely still lacks elucidation, however, the use of innovative technologies is improving our understanding [5]. Genome-wide association studies (GWAS) have identified novel genetic variants associated with differing traits such as early-onset CD (CTLA-4) [6,7].

The pathogenesis of IBD is multifactorial involving genetics, microbiota, and environment interacting with the host immune system [1,8,9]. Reflective of this complexity is the dynamic change with which different treatments alter both the host immune response and the gut microcosm [1,10,11]. For this reason, several treatment modalities exist, however, the taxonomic shift relative to treatment is still largely uncategorized [12]. Despite the differences between IBD subsets, both result from autoimmune tissue destruction, therefore, targeted therapy includes immunomodulators and immunosuppressants [3]. Tumor necrosis factor (TNF)-targeted treatment is effective but a large percentage of patients are incomplete or non-responders [13]. Just as the interaction between the gut microbiota and the host immune system is a factor in disease development, the changes in microbiomes throughout this progression may be related to the inadequate response [13].

Reduced inflammation in anti-TNF-treated patients results correspondingly from modulation of the gut microbiome which reverts to a composition comparable to that of healthy individuals including reduced Enterobacteriaceae coli (E. coli) and Ruminococcus species, as well as increased proportions of Bacteroidetes and Firmicutes [13]. Similarly, combinations of antibiotics have been clinically shown to improve flares in both adult and pediatric IBD patients likely by decreasing mucosal inflammation via the alteration of intestinal microbiota [14]. However, no single bacterial species have been implicated in disease causation, furthering that a non-specific imbalance coupled with other stressors lead to disease processes, including the less explored virome and fungal gut components [15-17].

Our capacity to evaluate the microbiome as a functional (or dysfunctional) component of the gastrointestinal system has impacted the way we can treat IBD including both antibiotics and probiotics, the latter intended to confer a beneficial population of bacteria often through dietary contribution for the relief of functional gut disorders [18-20]. A mainstay of IBD treatment is focused on immunosuppression such as in UC with the implementation of mesalamine, glucocorticoids, thiopurines, and biologic agents [21,22]. Despite this, immunosuppressive therapies to date are not known to sustain remission for UC patients [21].

The gut microbiome is seemingly involved in more than macromolecular digestion, as the pathogenic role of dysbiotic inflammation has been linked to other comorbidities such as mental illness, including depression [23,24]. Inflammatory cytokines and acute-phase proteins such as interleukin (IL)-6 and C-reactive protein (CRP) were evaluated through meta-analysis showing an increased incidence of depressive symptoms [23]. Co-morbid depressive symptoms predict poor disease course in IBD and are inconsistently improved by psychological treatments, inferring that disease progression including co-morbid depression may stem from the effects of treatment on gut flora [23,25]. Immunomodulators have shown meaningful changes in microflora populations with clinical improvement but more needs to be done to understand the impact of immunotherapy treatments on the gut microbiome to explain why these treatments may work in some patients but remain ineffective or partially effective in other patients. This systematic review explores the current literature on the association between immunotherapy and the microbiome of IBD patients.

## Review

Methods

Protocol

This systematic review was performed and reported in agreement with the Preferred Reporting Items for Systematic Reviews and Meta-Analyses (PRISMA) [26].

Inclusion/Exclusion Criteria

This literature search was performed to identify studies that defined immunotherapy and the gut microbiome in all-aged patients with IBD. The criteria used to search for eligible studies included the following: (1) response to immunotherapy in IBD, (2) variations in the gut microbiome in IBD, (3) variations in the gut microbiome in immunotherapy, and (4) variations in immunotherapy response in patients where the gut microbiome was studied. The studies that reported other illnesses or immunotherapy used in other illnesses were excluded as they were outside the scope of the extant study. The study types included were limited to randomized control trials (RCT), observational/non-randomized studies, and review articles either systematic in nature or unspecified.

Search Strategy

A methodical search of the PubMed [27], PubMed Central (PMC), and MEDLINE databases was conducted on April 29, 2021. The search for relevant studies using generic keywords (Immunotherapy) AND ((Gastrointestinal Microbiome) OR (Microbiota)) AND ((Inflammatory Bowel Disease) OR (Crohn Disease) OR (Ulcerative Colitis)) was done and 108 studies were identified. The relevant Medical Subject Headings (MeSH) terms and keywords "inflammatory bowel disease," "complications" "diet therapy" "drug therapy" "genetics" "immunology" "metabolism" "microbiology" "therapy" were used in various combinations using Boolean operators like "AND" and "OR," and 5,860 studies were identified. The inclusion/exclusion criteria were applied through an automation tool and records from January 2015 to April 2021 were identified. The search extended to studies published in the English language for human participants and returned 1,878 relevant studies. In accordance with the inclusion/exclusion criteria, gray literature was not included in this study. The results of the search strategy with respective keywords are displayed in Table 1 and Table 2.

**Table 1:** PubMed Database search results with regular keywords.

Keywords	Total Articles	Articles after Inclusion/Exclusion Criteria Applied by Automation
(Immunotherapy) AND ((Gastrointestinal Microbiome) OR (Microbiota)) AND ((Inflammatory Bowel Disease) OR (Crohn Disease) OR (Ulcerative Colitis))	108	32

**Table 2:** Displaying the entire MeSH search strategy. MeSH, Medical Subject Headings

MeSH	Total Articles	Articles after Inclusion/Exclusion Criteria Applied by Automation
Inflammatory Bowel Disease OR Crohn Disease OR Ulcerative Colitis ( "Inflammatory Bowel Diseases/complications"[Majr] OR "Inflammatory Bowel Diseases/diet therapy"[Majr] OR "Inflammatory Bowel Diseases/drug therapy"[Majr] OR "Inflammatory Bowel Diseases/genetics"[Majr] OR "Inflammatory Bowel Diseases/immunology"[Majr] OR "Inflammatory Bowel Diseases/metabolism"[Majr] OR "Inflammatory Bowel Diseases/microbiology"[Majr] OR "Inflammatory Bowel Diseases/therapy"[Majr] ) AND Immunotherapy ( "Immunotherapy/adverse effects"[Majr] OR "Immunotherapy/complications"[Majr] OR "Immunotherapy/drug effects"[Majr] OR "Immunotherapy/immunology"[Majr] OR "Immunotherapy/mortality"[Majr] OR "Immunotherapy/pharmacology"[Majr] OR "Immunotherapy/therapeutic use"[Majr] OR "Immunotherapy/therapy"[Majr] ) AND Gastrointestinal Microbiome OR Microbiota ( "Gastrointestinal Microbiome/drug effects"[Majr] OR "Gastrointestinal Microbiome/genetics"[Majr] OR "Gastrointestinal Microbiome/immunology"[Majr] )	5,707	1,878
((Inflammatory Bowel Disease OR Crohn Disease OR Ulcerative Colitis[MeSH Major Topic]) AND ( Immunotherapy[MeSH Major Topic])) AND (Gastrointestinal Microbiome OR Microbiot[MeSH Major Topic])	11	4
(("Inflammatory Bowel Disease/complications"[MESH]) AND "Immunotherapy/complications"[MESH] OR “Immunotherapy/adverse effects”) AND ( "Gastrointestinal Microbiome/drug effects"[MESH] )	6	2
((Inflammatory Bowel Disease[MeSH Terms]) AND (Immunotherapy[MeSH Terms])) AND (Gastrointestinal Microbiome[MeSH Terms])	19	9
(("Inflammatory Bowel Disease/complications"[MESH]) AND "Immunotherapy/complications"[MESH] OR "Immunotherapy/adverse effects") AND ( "Gastrointestinal Microbiome/immunology"[MESH] )	9	5

Data Extraction

Articles were screened by full-text, abstracts, and titles independently by two authors, KK and JA. Both parties reviewed and scrutinized the relevance, eligibility, and quality of the studies independently. Where necessary, differences in judgment were addressed through mutual discussion. 

Bias Evaluation and Data Explication

The quality appraisal was conducted to only include moderate-to-high quality studies in the final investigation and was conducted with the following tools: Cochrane Tool (randomized control trial), Newcastle-Ottawa checklist (observational/non-randomized control trial), and the Scale for the Assessment of Narrative Review Articles (SANRA) checklist (traditional review articles).

Results 

Search Outcome

**Figure 1:**
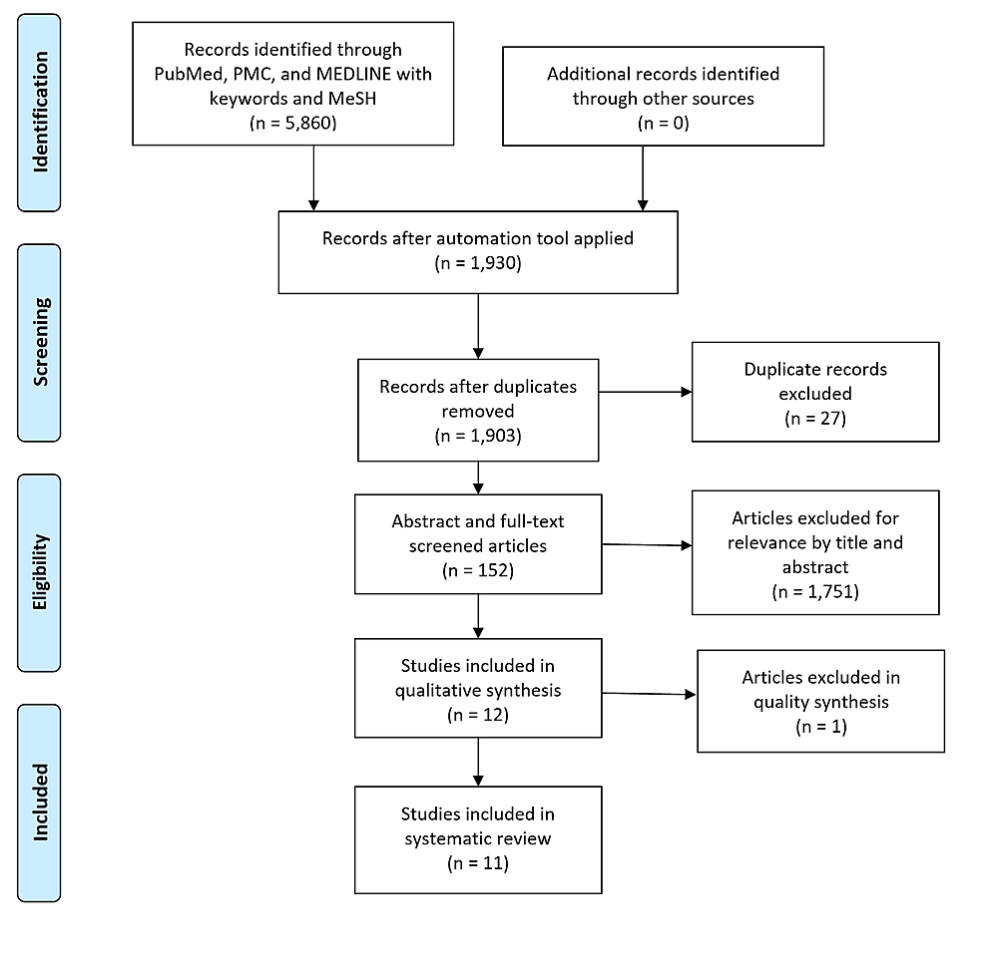
PRISMA flow diagram outlining the search process. PRISMA, Preferred Reporting Items for Systematic Reviews and Meta-Analyses; PMC, PubMed Central; MeSH, Medical Subject Headings; n, number of articles

A total of 5,860 papers were identified through a field search of PubMed, PMC, and MEDLINE. After the application of the inclusion/exclusion through the automation tool, 1,930 articles remained for further processing; 27 duplicates were removed using EndNote Basic and 1,903 articles were screened through title and abstract for relevance. 1,753 articles were excluded and the resulting 152 relevant articles were screened in-depth with the inclusion/exclusion criteria in the current analysis. A total of 12 relevant studies were assessed for quality appraisal and 11 moderate-to-high quality studies were included in this systematic review for discussion. Figure 1 demonstrates the PRISMA flow diagram in conducting the search for this review.

The 11 articles included in this review were published in English in peer-reviewed journals from 2015 to 2021 with full-texts freely available and discussed the interactions between the gut microbiome and immunotherapy in IBD patients. The included articles comprised observational/non-randomized control trials (n=6), review articles with unclear methods or traditional reviews (n=3), and RCT (n=2). One observational/non-randomized control trial was excluded on the basis of insufficient quality. The study findings of the included studies are summarized in Table 3 [3,28-37].

**Table 3:** Findings of the studies included in the analysis. Anti-TNF, anti-tumor necrosis factor; CyA, cyclosporin A; CD, Crohn's disease; IBD, inflammatory bowel disease; NOD2, nucleotide-binding oligomerization domain 2; CC, collagenous colitis; UC, ulcerative colitis; PROTECT, Predicting Response to Standardized Colitis Therapy; IL-33, interleukin-33; GPR43, G protein-coupled receptor 43; SCFA, short-chain fatty acid; KCD, Korean Crohn's disease; 16S rRNA, 16S ribosomal ribonucleic acid

Author	Year	Type of Study	Purpose of Study	Results
Sakurai et al. [28]	2020	Observational study	Evaluate mucosal microbiota and gene expression profiles associated with long-term remission after discontinuation of anti-TNF therapy.	Differences between gut bacterial communities were shown to be most pronounced between week 0 and week 24 of anti-TNF therapy. Treatment resulted in a shift in expression pattern which may be indicative of disease course.
O’Reilly et al. [29]	2020	Randomized control trial	Assess the effect of using colon-targeted delivery system CyA formulation on the composition and functionality of the gut microbiota.	Colon-targeted systems with or without CyA showed negligible effects on gut microbiota composition; the ex vivo colon model demonstrated relevance in predicting in vivo impact of drugs on the microbiota.
Li et al. [30]	2019	Observational study	Report an integrative analysis on CD-related genetic defects in innate immune function and the composition of the ileal microbiome by combining two consecutive patient cohorts.	Confirm significant effect of IBD phenotype, C. difficile infection, and NOD2 genotype on ileum-associated microbiota; additional IBD-related genotypes are associated with changes in ileal microbial composition.
Carstens et al. [31]	2019	Observational study	Characterize the microbiota of patients with CC compared with that of healthy controls and IBD patients.	Common mechanisms may underlie the pathogenesis of CC and IBD, as shifts in key taxa were similar; a specific fecal microbiome is seen in CC patients with active disease and corticosteroid treatment, while the microbiome of CC patients in remission resembled healthy controls.
Schirmer et al. [32]	2018	Observational study	Evaluate the role of the gut microbiome in disease course for new-onset, treatment-naïve, pediatric UC patients, as part of the PROTECT Study.	Baseline and longitudinal microbial trends involved in the progression of disease and remission were observed; there is evidence that the microbiome impacts treatment efficacy in UC and microbial biomarkers could inform treatment strategies.
Grigg and Sonnenberg [33]	2017	Review article	Literature review assessing the functional interactions between the immune system and the gut microbiota involved in inflammatory disease; further advocation for therapeutic modulation of host immune factors and therapeutic microbiota manipulation.	Gut microbiota impacts the quality of immune function and correspondingly, the immune system informs the geography and demographics of microbiota; therapies that modulate or re-establish beneficial interactions are promising treatments for inflammatory disease.
Guariso and Gasparett [34]	2017	Review article	Update on the recent advances in the treatment of pediatric IBD.	Many children with IBD do not respond to current treatment options which target known pathways of an intricate disease process; the development of reliable prognostic biomarkers is an essential leap towards tailored treatment.
Hodzic et al. [3]	2017	Review article	Literature review on the role of IL-33 in the regulation of intestinal immunity, involvement in the intestinal disease, and implication in potential therapeutics.	The role of IL-33 is context-dependent: its behavior in steady-state is different than in epithelial injury. IL-33 has a role in signaling within IBD that is still being understood and there is further need to recognize it within the context of potential immunomodulatory therapeutics.
Agus et al [35].	2016	Randomized control trial	Investigate the impact of high fat and high sugar diets on mice to better understand the mechanisms involved in modulation of host physiology within gut inflammation and microbiota composition and function compared to human IBD patients. Histological disease activity was measured using human CD mucosal biopsy specimens.	The high fat and high sugar diet created an inflammatory environment in the gut correlating with dysbiosis, however, the expression of GPR43 (SCFA receptor) was reduced in mice with this diet and is similarly reduced in CD patients compared with healthy controls.
Eun et al. [36]	2016	Observational study	Analyze the variation of intestinal microbial community structure in KCD patients by comparing fecal and mucosal tissue samples using metagenomic analysis with healthy controls.	Intestinal microbial community structure in KCD patients was shown to be similar to that of Western CD patients as demonstrated by 16S rRNA sequencing anti-TNF treatment may affect microbial community structure.
Lewis et al. [37]	2015	Observational study	Analyze fecal samples with shotgun metagenomics from a prospective cohort of pediatric CD patients starting therapy with enteral nutrition or anti-TNFα antibodies to assess the full complement and dynamics of the microcosm during treatment.	Dysbiosis in CD also includes aberrations in fungi composition and the nature of dysbiosis is unique to individual stressors; the extent, however, diminished with reduction of inflammation by treatment including anti-TNF.

Discussion 

We studied 11 previously published articles to understand the relationship between human gastrointestinal tract microbial populations and the use of immunotherapy in IBD-afflicted patients. In this review, we aimed to gain an updated understanding of this relationship and how disease recurrence or remission is achieved inconsistently among patients. 

Microbiome Aberrations in Diseased and Healthy States

The pathogenesis of IBD is multifactorial but the gut microbiome is a significant component of chronic inflammatory disorders as shown by the mechanistic understanding of current treatments and documented population shifts. A study conducted by Carstens et al. compared fecal samples between patients with collagenous colitis (CC) and patients with CD and UC in addition to healthy controls [31]. Their findings demonstrated lower microbial diversity among CC patients as compared to healthy controls which resembled the findings of IBD patients, including a shift in the Ruminococcaceae family (a member of the Firmicutes phylum) [31]. Interestingly, a study conducted by Eun et al. observed that Firmicutes populations declined in IBD-afflicted patients in both Korean and Western subjects despite their differences in dietary habits (including proinflammatory high fat and confectionary diets typical in Western cuisine) which are known to affect intestinal microorganisms [35,36]. Beneficial host-microbiota interactions rely on multiple factors including diet and systemic immunoglobulin responses [33]. The role of Firmicutes, in particular, is implicated in gut health as an energy source and inflammatory modulation via fermentation of dietary fiber to produce important short-chain fatty acids (SCFA) such as acetate, propionate, and butyrate [36]. Patients with IBD are exposed to both dietary changes and antibiotics which invariably alter microbial community structures, although the precise effects are not completely understood [37]. Despite relative similarities between microbial demographics in CC as compared with IBD, they are phenotypically different diseases. Furthermore, while dysbiosis commonly refers to bacterial microbial structures, other domains within the gut microbiome are less studied and may contribute to unaccounted variation between patients [37].

Similarly, Eun et al. compared intestinal microbial communities in Korean CD (KCD) patients with that of Western CD patients and healthy controls [36]. Their deep sequencing study demonstrated dysbiosis patterns in the KCD patients that were comparable to their Western counterparts, including decreased microbial diversity and proportionately increased Proteobacteria and Fusobacteria populations while Firmicutes and Bacteroides populations declined [36]. Importantly, the microbial demographics in this study differed from the healthy controls where both mucosal tissue and fecal samples showed Escherichia and Shigella species abundantly in the diseased patients which coincide with known literature involving the role of E. coli strains in inducing inflammation [35,36].

A study by Lewis et al. used shotgun metagenomic sequencing to analyze fecal samples from a prospective cohort of CD-afflicted pediatric patients beginning either enteral nutrition or anti-TNFα antibodies and observed that dysbiosis occurred through not only bacterial composition but also differences in gene representation, higher fungal expanse, and increased volume of human DNA in the stool [37]. They further discuss that the independent effects of antibiotics, diet, and inflammation are reflective of mechanistic differences and, although dysbiosis in CD is common, the quality of these aberrations is unique to the stressor [37]. However, there has been conflicting evidence surrounding UC and microbial diversity which has sometimes been indifferent compared with healthy individuals [31].

The relative success of fecal microbial transplant (FMT) is considered low or inconclusive at present, with minimal data to support recommendations for IBD treatment, however, ongoing medical management is aimed at modulating inflammation and altering microbiome expression [34]. Multiple studies have demonstrated that the diminished diversity of taxa in inflammatory disease states often have similar profiles. While it might be expected that similar microbial profiles would produce a similar disease phenotype, perhaps finding differences in the overall microcosm profiles (including viruses and fungi) between disease states or other host differences such as genetic polymorphisms may explain differing disease phenotypes under similar aberrant microbial profiles.

Gene Expression Implicated in Disease

Gene expression in both the host and the gut microcosm interact with environmental stressors to create a favorable (or unfavorable) condition for the development of inflammatory autoimmune diseases. A study by Sakurai et al. evaluated the association between long-term remission in UC after discontinuation of anti-TNF therapy, mucosal microbiota, and gene expression profiles [28]. They used machine learning with IBD GWAS data as a predictive tool in a similar method as is used to predict cancer susceptibility, recurrence, and survivability [28]. UC follows a relapsing and remitting course where machine learning, as a predictive tool, could advise patients on therapy discontinuation [28]. They found that gene expression in the non-relapse UC group had substantially higher gene expression of ALIX, also known as programmed cell death 6-interacting protein (PDCD6IP), and ​​​​SLC9A3(Solute carrier family 9 member A3) compared with the relapse group [28]. Furthermore, it is speculated that expression of ALIX, IL22RA (interleukin-22 receptor subunit alpha-1), LGR6 (leucine-rich repeat-containing G protein-coupled receptor 6), and SLC9A3 may prevent UC flares since these genes were downregulated after anti-TNF therapy in the relapse group while in heat shock proteins and ITGA7 (integrin alpha 7) were upregulated in this group [28].

Another study by Li et al. explored abnormal host-microbial interactions through genetic susceptibility, focusing on CD-related genetic defects in innate immune function and specifically the ileal microbiome by comparing phenotypic variants of CD comprised of ileum-affected, colitis, or non-IBD patients [30]. This group’s previous work examined nucleotide-binding oligomerization domain-containing protein 2 (NOD2) and protein-coding gene, autophagy-related 16-like 1 (ATG16L1), polymorphisms on ileal microbial populations including the link between NOD2 genotypes and C. difficile infection [30]. Further, differential gene expression altering Paneth cell function was found to promote dysbiosis [30]. In the current study, higher representation of the Proteobacteria phylum was linked to the NOD2R genotype independent of IBD phenotype even in disease unaffected ileal mucosa and may be related to the observation of relatively lower Proteobacteria abundance in unaffected mucosa compared with adjacent disease affected mucosa in ileal CD phenotypes [30]. The differences between these mucosal neighborhoods strengthen the concept of dysbiosis in normal-appearing mucosa [30].

Genetic susceptibility may vary among different ethnic populations. Susceptibility loci differ between Asian and Western populations and while NOD2 and ATG16L1 do not show replication results in Asian populations, the incidence of CD in Asian populations is increasing [36]. ATG16L2 mutations have been observed in KCD patients and are known to regulate bacterial signaling, which (although different from the Western mutations), is similarly involved in bacterial modulation which reinforces the concept that abnormal interactions between gut microflora and susceptible host genes promote CD development [36]. IBD phenotype and genetic variation underly, in part, how the host immune system interacts with local flora; furthermore, although certain identified genes can help predict remission, a methodology for how these factors interact with therapy needs further elucidation. Machine learning provides a hopeful framework for a predictive approach to disease course but further work on the mapping of the gut microbiome, genetic variability, and their interaction with therapy is needed to provide solutions for disease recurrence and treatment non-responders.

Interactions between the Microbiome and Immunotherapy

The use of both probiotics and antibiotics are broadly used to treat gastrointestinal disorders by the targeted alteration of microbial populations, however, microbial communities at the intestinal level have been shown to change throughout immunomodulation in IBD by either direct or indirect causes. Intestinal microbiota can contribute to xenobiotic metabolism which may influence drug bioavailability and ultimately the efficacy and safety of oral medications [29]. A study by O’Reilly et al. evaluated a colon-targeted delivery system (encapsulated SmPill minispheres) for cyclosporine A (CyA) on the composition and functionality of gut microbiota [29]. Steroid-refractory UC can be treated with systemic CyA and infliximab, however, CyA has a narrow therapeutic window and requires monitoring [29]. In this *ex vivo* colon model (and the conducted *in vivo pilot *of healthy volunteers), gut microbiota composition was not affected but the impact of immunosuppression on the host as a downstream influence on microbiota could not be ruled out [29]. Furthermore, SmPill formulations increased the concentration of n-butyrate, which is known to positively correlate with host health [29].

A lack of immunosuppressant maintenance treatment and poor mucosal healing is associated with an increased risk of relapsing in UC patients who discontinue anti-TNF therapy [28]. The maintenance of gut homeostasis is, in part, maintained by Dore sp. and Lachnospira sp. genera, which produce beneficial SCFAs [28]. The Sakurai et al. study reported that gut microbial surveillance at week 0 and 24 post-anti-TNF therapy cessation are predictive of sustained remission after initial anti-TNF therapy remission [28]. Conversely, pro-inflammatory Fusobacterium sp. and Veillonella dispar are associated with relapse [28].

Immune education, development, and response are impacted by the microbiome and its products. As part of the PROTECT study, Schirmer et al. evaluated taxonomic shifts on treatment efficacy of treatment naïve, pediatric UC patients [32]. They found that patients with severe disease, compared with their baseline, had higher populations of oral cavity bacteria than expected which infers that severe disease may correlate with a propensity of aberrant colonization compared with resistant, healthy guts [32]. Expression of virulence genes by microbes may also aid in abnormal colonization and exacerbate inflammation including antibiotic resistance and adhesion mechanisms [32].H. Parainfluanzaewas particularly identified on biopsy as an active constituent in UC patients, correlating with disease severity and treatment efficacy [32]. Specifically, in CD treated with infliximab, as in the Eun et al. study, the abundance of Gammaproteobacteria was proportionally increased in both fecal and mucosal tissue samples [36]. Some animal models suggest the microbiome causes an adjuvant effect that primes autoreactive adaptive immune responses even in extraintestinal sites [33]. Whether microbial participation is a cause or consequence of UC, it is innately tied to the pathophysiology of IBD as microbial biomarkers are also predictive of treatment efficacy and may have a trajectory in furthering machine learning as a predictive tool, as described by Sakurai et al. [28,32] Further work to understand microbiome profiles in both diseased and healthy states may elucidate our understanding of treatment non-responders, as intestinal-level metabolism impacts drug bioavailability [29].

Limitations

There are some limitations to this study as only work published in the English language in the past six years involving human subjects was evaluated. We may have missed studies published in other languages outside of this time frame, which may impact the data available to us. Both the genetic constitution and dietary habits of the human population have been shown to impact the development of IBD and gut microbial communities, therefore, we may have missed other valuable studies published in different languages which may assess this topic in different cultural populations. This study focused on the involvement of human subjects, however, exclusively animal models not included here may provide further insight into disease process and treatment.

## Conclusions

In this systematic review, we aimed to compile information on the interactions of the gut microbiome with immunotherapy in the course of disease and treatment of IBD patients. After thoroughly reviewing the studies, we found that although there are characteristic taxonomic shifts between diseased and healthy patients, genetic variants are an important consideration in the predictive quality of disease and treatment decisions. Despite genetic and dietary factors, the characteristic shifts in microbial populations were similar among the studied Asian and Western groups. Furthermore, the bioavailability of some treatments may be impacted by existing intestinal microbes; while intestinal microbial populations may change directly or indirectly in the course of treatment.

The juxtaposition between the interaction of microbial populations and treatment in addition to the existence of genetic variants may provide insight into why some patients do not respond fully to therapy. We believe that further categorization of microbial changes between diseased and healthy states will improve our therapeutic potential and prevention of both gastrointestinal and multi-system diseases. Limitations in our current knowledge including the non-bacterial components of the gut microcosm and the complexity of both the metagenomic gut composition and genetic variants of IBD need further clarity to improve our clinical predictive tools. Our study is necessary for furthering the need for specific characterization of microbial changes in IBD processes and treatment. Future studies should focus on the comparison between Western and other cultural populations as well as further implementation of GWAS in clinical predictability.
